# Association between metabolic syndrome and hip osteoarthritis in middle-aged men and women from the general population

**DOI:** 10.1371/journal.pone.0230185

**Published:** 2020-03-10

**Authors:** Sven S. Walter, Elke Wintermeyer, Christian Klinger, Roberto Lorbeer, Wolfgang Rathmann, Annette Peters, Christopher L. Schlett, Barbara Thorand, Sergios Gatidis, Konstantin Nikolaou, Fabian Bamberg, Mike Notohamiprodjo

**Affiliations:** 1 Department for Diagnostic and Interventional Radiology, Eberhard Karls University Tuebingen, University Hospital Tuebingen, Tübingen, Germany; 2 Department for Trauma and Reconstructive Surgery, BG Trauma Center Tuebingen, Eberhars Karls University Tuebingen, Tuebingen, Germany; 3 Department of Radiology, Ludwig-Maximilian-University Hospital Marchioninistraße, Munich, Germany; 4 German Center for Cardiovascular Disease Research, Munich, Germany; 5 Department of Biometry and Epidemiology, German Diabetes Center, Düsseldorf, Germany; 6 Institute of Epidemiology, Helmholtz Zentrum München, German Research Center for Environmental Health, Neuherberg, Germany; 7 Institute for Cardiovascular Prevention, Ludwig-Maximilian-University Hospital, Munich, Germany; 8 Department of Diagnostic and Interventional Radiology, Medical Center ‐ University of Freiburg, Freiburg, Germany; University of Tasmania, AUSTRALIA

## Abstract

**Objective:**

To investigate the impact of metabolic syndrome and its components on osteoarthritis of the hip joints compared to a healthy cohort in the KORA MRI-study.

**Methods:**

Randomly selected men and women from the general population were classified as having metabolic syndrome, defined as presence of central obesity plus two of the following four components: elevated blood pressure (BP), elevated fasting glucose, elevated triglycerides (TG) and low HDL-cholesterol (HDL-c), or as controls without metabolic syndrome. Therefore, each subject underwent detailed assessment of waist circumference as well as fasting glucose, systolic and diastolic BP, TG, and HDL-c concentrations as well as a full-body MR scan. MR measurements were performed on a 3 Tesla scanner (Magnetom Skyra, Siemens) including a dual-echo Dixon and a T2 SS-FSE sequence for anatomical structures. In order to quantify osteoarthritis of the hip, assessment was performed by two independent, experienced radiologists for joint gap narrowing, osteophytic lipping and subchondral changes (e.g. sclerosis, pseudocysts). Associations between metabolic syndrome components and hip degeneration were estimated by logistic regression models providing odds ratios.

**Results:**

Among 354 included participants (mean age: 56.1 ± 9.2 years; 55.4% male), 119 (34%) had metabolic syndrome, while 235 (66%) were part of the control group. Except for elevated blood glucose (p = 0.02), none of the metabolic syndromes’ component was independently associated with osteoarthritis. Multivariable adjusted ORs for osteoarthritis of the right hip were 1.00 (95% CI 0.98;1.03), 1.00 (95% CI 0.99;1.00), 1.01 (95% CI 0.99;1.03), 1.00 (95% CI 0.97;1.04) and 1.01 (95% CI 0.96;1.06), and for the left hip 1.00 (95% CI 0.98;1.03), 1.00 (95% CI 1.00;1.01), 1.01 (95% CI 0.99;1.03), 0.99 (95% CI 0.96;1.02) and 1.04 (95% CI 0.99;1.09) for waist circumference, triglyceride, HDL-c and systolic and diastolic BP, respectively. Blood glucose was a borderline non-dependent factor for osteoarthritis of the right hip (OR: 1.02 (95% CI 1.0;1.04); p = 0.05). Furthermore, the compound metabolic syndrome was not significantly associated (OR left hip: 1.53 (95% CI 0.8;2.92), p = 0.20; OR right hip: 1.33 (95% CI 0.72;2.45), p = 0.37) with osteoarthritis of the hip joint. Age as well as gender (left hip) were the only parameters in univariate and multivariate analysis to be significantly associated with osteoarthritis of the hip joint.

**Conclusion:**

The compound metabolic syndrome showed no association with osteoarthritis of the hip joint. Age was the only parameter to be dependently and independently associated to osteoarthritis of both hip joints, while elevated blood glucose was independently associated with degeneration of the right hip joint.

## Introduction

Osteoarthritis and metabolic syndrome are two of the leading disorders in developed countries [1–5]. Metabolic syndrome, a constellation of hazardous factors leading to diabetes mellitus as well as to cardiovascular disorders, contains common risk factors for both diseases such as increased blood glucose levels, elevated waist circumference (central obesity), elevated blood pressure, increased triglycerides and lowered HDL-c [5–10].

Osteoarthritis is a major cause for a reduction in quality of life due to restriction in activity and disability, without a curative treatment option and the ultima ratio being total joint replacement [9, 11–13]. The prevalence is estimated to be between 30–50%, leading to stiffness, general joint pains, reduced range of motion and increased morbidity and mortality [1, 4, 14]. Osteoarthritis and metabolic syndrome are often co-existent, whereby the direct impact on each other remains for the most parts unclear. There are multiple risk factors for developing osteoarthritis of the weight bearing joints (e.g. knee or hip joint), such as age, nutritional habits or central obesity, which are concomitantly responsible for causing or worsening diabetes [1, 4, 8, 10, 15, 16]. Yet, osteoarthritis and metabolic syndrome may simply be co-existing through these common risk factors [9, 17].

On a pathophysiological level, it is believed that adipose tissue and chondrocytes produce adipokines, which regulate adipocyte metabolism, the immune- and inflammatory response. These adipokines were identified in osteoarthritic joints and in vitro to produce comparable chondrocyte activation as seen in proinflammation and mechanical stress [15, 16, 18, 19]. Furthermore, high levels of glucose may disturb the homeostatic state of chondrocytes due to multiple mechanisms such as failed regulation of glucose transporters (GLUT-1) receptors or accumulated advanced glycation products [18]. Latest investigations suggest, that not only overweight of patients with metabolic syndrome plays a major role in the pathophysiology of osteoarthritis but also other components, especially hypertension [1, 3, 11, 18, 20]. Hypertension is thought to reduce the blood flow to subchondral microvessels, thus, reducing the supply of nutrients and oxygen, ultimately leading to ischemia [18]. In addition, lipid disorders are believed to influence the metabolism of chondrocytes, thus, interfering with joint metabolism [21, 22].

The present analysis based on data from the population-based KORA study conducted in Augsburg, Southern Germany, may help to further elucidate mechanisms linking the metabolic syndrome with osteoarthritis of the hip, resulting in metabolic osteoarthritis. Thus, the purpose of this study was to investigate the impact of metabolic syndrome and its components on osteoarthritis of the hip joints compared to a healthy cohort in the KORA MRI-study.

## Material and methods

### Study design

The prospective KORA-Study (Cooperative Health Research in the Augsburg Region) is a research platform build on several population-based surveys conducted within the study region. Therefore, cities were cluster sampled and participants between 25 and 74 years were stratified randomly selected. Details have been previously described elsewhere [23]. The present analysis is based on the KORA MRI substudy. Participants were selected from the 2^nd^ KORA follow up FF4 between 2013 and 2014 (baseline survey 1999–2001), consisting of 400 individuals [24]. To be included in the MRI substudy, the participants had I) to be willing to undergo whole-body MRI, II) full health assessment, III) to be ≤ 72 years old and IV) no known contraindication for MRI exams (e.g. pace maker) [23].

The exclusion criteria for the present analysis were I) hip joints not assessable due to artifacts or hip joint not fully illustrated, II) hip joint replacement and III) missing laboratory results.

This study was approved by the institutional review board of the Ludwig Maximilian’s University Munich (Germany) and written consent was obtained from each participant.

### Metabolic syndrome

At the FF4 study visit, all participants underwent standard assessment for metabolic syndrome, which was defined, as suggested by the international diabetes foundation (IDF), as the presence of central obesity plus two of the following four components: elevated blood pressure (BP), elevated fasting glucose, elevated triglycerides and low HDL-cholesterol [7].

For the evaluation of central obesity, waist circumference was measured (central obesity: ≥94 cm [males]; ≥80 cm [females]).

Elevated BP was defined as systolic blood pressure ≥ 130 mmHg and/or diastolic blood pressure ≥ 85 mmHg, or self-reported antihypertensive drug treatment [7].

Elevated fasting glucose was defined as values ≥100 mg/dl or previously diagnosed diabetes mellitus with use of antidiabetic medication [7].

Raised serum triglycerides were defined as ≥ 150 mg/dl or drug treatment for elevated triglycerides, while low HDL-C (high density lipoproteins) was defined as < 40 mg/dl [males]; < 50 mg/dl [females] [7].

### Covariates

Not included in the IDF definition, we evaluated the body mass index (BMI) using the subjects’ weight in kilogram (kg) divided by the square of the height (m^2^).

Physical activity was assessed according the regularity and the active hours per week (none/almost none; approximately 1 hour/week on an irregular basis; regularly about 1 hour/week; regularly over 2 hours/week).

All participants were assessed for their smoking status and classified in three groups, never smoker, ex-smoker or active smoker.

### MR imaging protocol

Whole-body MR measurements were performed on a 3 Tesla scanner (Magnetom Skyra, Siemens Healthcare, Erlangen, Germany). Description in detail of technical and imaging protocols are described elsewhere [23]. For assessing anatomical structures, a transversal dual-echo Dixon and a coronal T2w single shot fast spin echo (SS-FSE/HASTE) sequence were employed. Imaging parameters dual-echo Dixon: 256 x 256, field of view (FOV): 488 x 716 mm, echo time (TE) 1.26 ms and 2.49 ms, repetition time (TR): 4.06 ms, partition segments: 1.7 mm, flip angle: 9°. Image parameter T2 Haste: matrix: 320 x 200, field of view (FOV): 296 x 380 mm, echo time (TE) 91 ms, repetition time (TR): 1000 ms, partition segments: 5 mm, flip angle: 131°.

### Image analysis of hip osteoarthritis

All participants were anonymized, blinded and read randomly. Evaluation was individually performed by two radiologists with 3 (SW) and 11 years (MN) of experience in musculoskeletal imaging. In order to perform quality control as well as intrareader agreement, 40 participants were reevaluated in a blinded and randomized fashion by the primary reader.

To quantify osteoarthritis of the hip, assessment was performed on an osteoarthritis MRI score (OA MRI score), which was based on the Kellgren-Lawrence classification [25]. Subchondral changes (sclerosis, pseudocysts) were assessed on a 4-point scale (0 = none, 1 = little, 2 = moderate with cysts, 3 = heavy with cysts). Osteophytic lipping was evaluated on a 3-point scale (0 = none, 1 = small, 2 = large).

**Fig 1:**
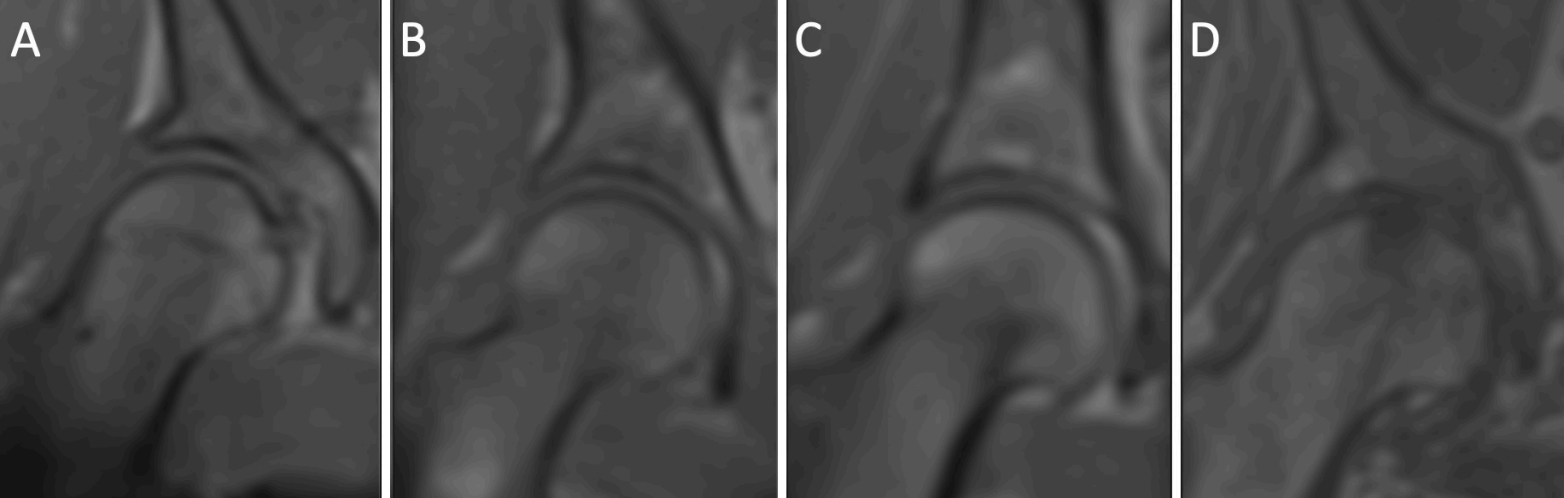
Examples of T1 vibe dixon in phase of the degenerative states of the OA MRI score. A: Grade 0 –no degeneration; B: Grade 1 –Slight narrowing of the medial joint gap and small osteophytic lipping; C: Grade 2 –narrowing of the medial joint gap and increased osteophytic lipping; D: Grade 3 –loss of the medial joint gap, osteophytic lipping as well as pseudocystic lesions.

Furthermore, the gap of the hip joints was measured cranially and medially (in mm). The mean of the measured cranial and medial joint gap was calculated and classified into a 4-point scale in order to evaluate the narrowing of the joint (0 = none/questionable [≥4 mm], 1 = narrowing [2–4 mm], 2 = heavy narrowing [0.1–2 mm], 3 = loss of joint gap [0 mm]). The evaluated points were added and graded as followed–Grade 0 = 0 points; Grad 1 = 1–2 point(s); Grade 2 = 3–4 points; Grade 3 = 5–7 points and Grade 4 = 8 points (Fig 1). Grad 1 and above was defined as pathological.

### Statistics

Risk factor characteristics and osteoarthritis status of study participants were summarized separately for those with and without the metabolic syndrome using mean and standard deviation for continuous measurements and absolute numbers and percent values for categorical measurements. Differences among study groups were evaluated by two-sided t-test, χ^2^-test or Fisher’s exact test.

Correlations and associations of metabolic syndrome components with degeneration of the left and right hip were estimated by univariate and multivariable adjusted logistic regression models providing odds ratios (OR) with 95% confidence interval (CI), respectively. Multivariable adjusted model A included age, sex, medication and single components of the metabolic syndrome (waist circumference, triglyceride, HDL-C, BP, and glucose) on a continuous scale whereas model B included age, sex and dichotomized single components of the metabolic syndrome (obesity, elevated triglycerides, low HDL-C, elevated BP, and elevated glucose) and model C included age, sex and the overall combined variable for the metabolic syndrome. A p-value of <0.05 was considered statistically significant. To account for multiple testing of the six multivariable logistic regression models with metabolic risk factors as continuous (model A), dichotomized (model B) and combined (model C) independent variables and left and right hip degeneration as dependent outcome variables, respectively, we additionally evaluated the results based on a significance level of p<0.008 (0.05/6). For the evaluation of inter- and intrareader agreement of hip degeneration measurements Cohen’s Kappa (K) was used and agreement was defined as follows: 0.01–0.20 as poor, 0.21–0.40 as fair, 0.41–0.60 as moderate, 0.61–0.80 as substantial and 0.81–1.0 as almost perfect [26]. Statistical analyses were performed using Stata 14.1 (Stata Corporation, College Station, TX, U.S.A.).

## Results

### General results

**Fig 2:**
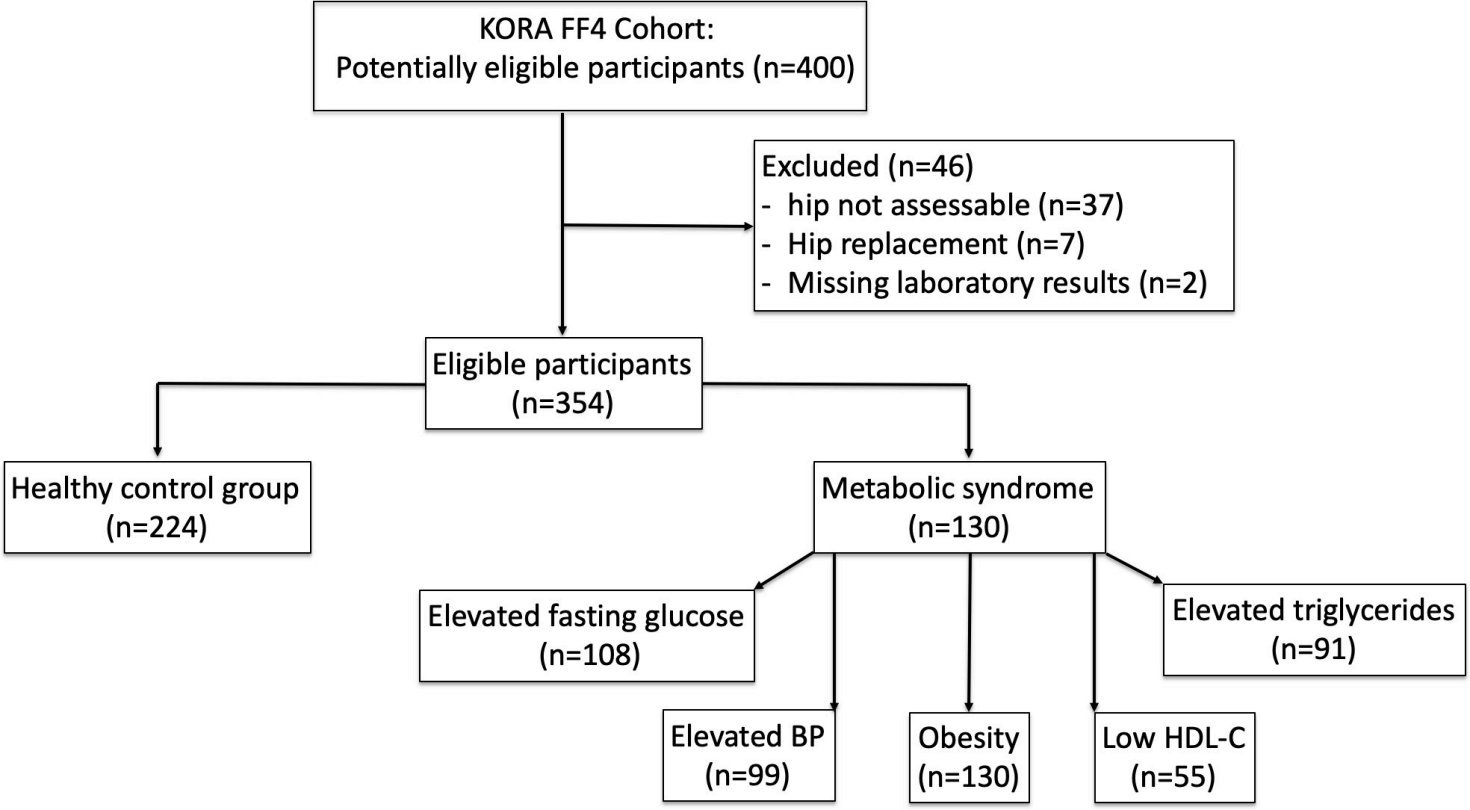
Inclusion flow chart.

Out of the 400 participants of the KORA-MRI Study, 354 (89%) subjects were included in the present analysis. Forty-six (11.5%) subjects met the exclusion criteria due to none assessable image quality (9.3%), hip replacement (1.8%) of whom all had metabolic syndrome, and missing laboratory results (0.5%). Of the included subjects, 130 (36.7%) had metabolic syndrome while 224 (63.3%) were part of the healthy control group (Fig 2).

Patient demographics and descriptive patient data about metabolic risk factors are given in Table 1. Patients with metabolic syndrome were mostly male (67.7%), obese with an elevated BMI and waist circumference as well as a higher prevalence of elevated BP compared to the control group (p<0.001). Furthermore, HbA1c and triglycerides were significantly higher, while HDL was significantly lower (p<0.001). The controls were physically more active, however, group differences were not significant (p = 0.07). In addition, the prevalence of the smoking status showed no significant difference between participants with metabolic syndrome and the controls (p = 0.18).

**Table 1:** Demographics and risk factors for hip degeneration.

Characteristics	Entire Cohort	Metabolic Syndrome	Controls	p
	N = 354	N = 130	N = 224	
Age (years)	56.1 ± 9.3	58.7 ± 8.4	54.6 ± 9.4	<0.001
Gender (men)	196 (55.4%)	88 (67.7%)	108 (48.2%)	<0.001
Obesity	260 (73.5%)	130 (100%)	130 (58%)	<0.001
BMI (kg/m^2^)	28 ± 5	31.1 ± 4.7	26.2 ± 4.3	<0.001
Waist circumference (cm)	97.9 ± 14,5	108.1 ± 12.1	92 ± 12.5	<0.001
Elevated BP	150 (42.4%)	99 (76.2%)	51 (22.8%)	<0.001
Systolic BP (mmHg)	120.2 ± 16.7	128.7 ± 17.4	115.3 ± 14.1	<0.001
Diastolic BP (mmHg)	75.2 ± 10	79.2 ± 10.8	72.8 ± 8.7	<0.001
Elevated glucose	161 (45.5%)	108 (83.1%)	53 (23.7%)	<0.001
HbA1c (%)	5.5 ± 0.6	5.8 ± 0.8	5.4 ± 0.3	<0.001
HbA1c (mmol/)	37.1 ± 6.4	40.2 ± 8.4	35.2 ± 3.7	<0.001
Low HDL-cholesterol	71 (20.1%)	55 (42.3%)	16 (7.1%)	<0.001
HDL-C (mg/dl)	62.6 ± 17.6	55.2 ± 15.9	66.9 ± 17.1	<0.001
LDL-C (mg/dl)	139.6 ± 32.8	142.3 ± 33.3	138.1 ± 32.4	0.24
Elevated triglycerides	120 (33.9%)	91 (70%)	29 (13%)	<0.001
Triglycerides (mg/dl)	129.3 ± 81.7	176.7 ± 102.5	101.8 ± 49.1	<0.001
Physical activity				0.08
≥2h per week, regularly	100 (28.3%)	30 (23.1%)	70 (31.3%)	
1h per week, regularly	113 (31.9%)	37 (28.5%)	76 (33.9%)	
1h per week, unregularly	52 (14.7%)	22 (16.9%)	30 (13.4%)	
No or nearby no activities	89 (25.1%)	41 (31.5%)	48 (21.4%)	
Smoking status				0.18
Never smoker	130 (36.7%)	41 (31.5%)	89 (39.7%)	
Ex-smoker	147 (41.5%)	62 (47.7%)	85 (38%)	
Current smoker	77 (21.8%)	27 (20.8%)	50 (22.3%)	
*Degeneration*				
OA MRI score				
Left				0.19
Grade 0	61 (17.2%)	17 (13.1%)	44 (19.6%)	
Grade 1	272 (76.8%)	107 (82.3%)	165 (73.7%)	
Grade 2	21 (5.9%)	6 (4.6%)	15 (6.7%)	
Grade 3	0 (0%)	0 (0%)	0 (0%)	
Right				0.06
Grade 0	67 (18.9%)	20 (15.4%)	47 (21%)	
Grade 1	271 (76.6%)	108 (83.1%)	163 (72.8%)	
Grade 2	15 (4.2%)	2 (1.5%)	13 (5.8%)	
Grade 3	1 (0.3%)	0 (0%)	1 (0.5%)	
Hip Degeneration				
Left	293 (82.8%)	113 (86.9%)	180 (80.4%)	0.12
Right	287 (81.1%)	110 (84.6%)	177 (79%)	0.20

Data are given as mean ± standard deviation or number (percentage)

p-values are from t-test, chi^2^- test or Fisher’s exact test

### Osteoarthritis

In total, the prevalence of osteoarthritis was 88.7% (314/354), with a prevalence for the right and left hip joint of 81.1% and 82.8%, respectively. In regards to the metabolic syndrome, there was no significant difference in the prevalence of osteoarthritis of the hip joints between those with and without the metabolic syndrome (p≥0.06; Table 1). Furthermore, there was no significant difference between the group with metabolic syndrome and controls regarding each criterion of the OA MRI score, with osteophytes being borderline non-significant (p = 0.05).

In univariate as well as multivariable analysis, age was significantly associated with osteoarthritis (p≤0.01) (Tables 2 and 3). Furthermore, men showed significantly lower degeneration of the left hip in univariate analysis (p = 0.04), which was also confirmed in multivariable analysis when analyzing participants with metabolic syndrome (p = 0.03; Model C). However, when testing for each component in multivariable analysis (Model B) both hip joints showed significantly decreased osteoarthritis in men (p≤0.04; Tables 2 and 3).

**Table 2:** Unadjusted ORs (95% CI) for associations of continuous individual metabolic syndrome components with hip degeneration.

	Hip Degeneration			
Risk factors	Left		Right	
	OR (95% CI)	p	OR (95% CI)	p
Age (years)	1.06 (1.02; 1.09)	0.001	1.05 (1.02; 1.09)	0.001
Gender (men)	0.55 (0.31; 0.98)	0.04	0.64 (0.37; 1.10)	0.11
Waist circumference (cm)	1.00 (0.98; 1.02)	0.93	1.00 (0.98; 1.02)	0.83
Systolic BP (mmHg)	1.01 (0.99; 1.02)	0.47	1.01 (0.99; 1.02)	0.40
Diastolic BP (mmHg)	1.01 (0.98; 1.04)	0.41	1.00 (0.98; 1.03)	0.74
Fasting Glucose (mg/dl)	1.00 (0.99; 1.02)	0.57	1.02 (1.00; 1.04)	0.05
HDL-C (mg/dl)	1.01 (1.00; 1.03)	0.08	1.01 (1.00; 1.03)	0.11
TG (mg/dl)	1.00 (1.00; 1.00)	0.93	1.00 (1.00; 1.00)	0.21
Metabolic syndrome	1.62 (0.89; 2.98)	0.12	1.46 (0.82; 2.59)	0.20

**Table 3:** Multivariable adjusted OR (95% CI) for associations of metabolic syndrome components with hip degeneration. Model A: Analysis of each component of the metabolic syndrome on a continuous scale. Model B: Association of the single metabolic syndrome components with osteoarthritis. Model C: Analysis of each participant suffering from metabolic syndrome regardless each component.

	Hip Degeneration			
Risk factors	Left		Right	
	OR (95% CI)	p	OR (95% CI)	p
*Model A*				
Age (years)	1.06 (1.02; 1.10)	0.003	1.04 (1.01; 1.08)	0.002
Gender (men)	0.53 (0.26; 1.09)	0.09	0.60 (0.30; 1.21)	0.15
Waist circumference (cm)	1.00 (0.98; 1.03)	0.84	1.00 (0.98; 1.03)	0.81
Systolic BP (mmHg)	0.99 (0.96; 1.02)	0.52	1.00 (0.97; 1.04)	0.88
Diastolic BP (mmHg)	1.04 (0.99; 1.09)	0.15	1.01 (0.96; 1.06)	0.62
Fasting Glucose (mg/dl)	1.00 (0.98; 1.02)	0.95	1.02 (0.99; 1.04)	0.20
HDL-C (mg/dl)	1.01 (0.99; 1.03)	0.26	1.01 (0.99; 1.03)	0.59
TG (mg/dl)	1.00 (1.00; 1.01)	0.44	1.00 (0.99; 1.00)	0.19
Antihypertensive medication	0.97 (0.42; 2.23)	0.94	1.14 (0.52; 2.53)	0.74
Lipid-lowering medication	0.87 (0.26; 2.84)	0.81	0.83 (0.26; 2.66)	0.76
Anti-diabetic medication	1.29 (0.25; 6.66)	0.76	0.85 (0.14; 5.33)	0.86
*Model B*				
Age (years)	1.05 (1.01; 1.09)	0.01	1.05 (1.01; 1.08)	0.01
Gender (men)	0.47 (0.25; 0.88)	0.02	0.53 (0.29; 0.97)	0.04
Obesity	0.86 (0.44; 1.69)	0.67	1.10 (0.59; 2.08)	0.76
Elevated BP	1.23 (0.64; 2.37)	0.54	0.94 (0.50; 1.77)	0.85
Elevated glucose	1.49 (0.78; 2.85)	0.23	2.13 (1.12; 4.06)	0.02
Elevated triglycerides	1.20 (0.58; 2.49)	0.63	1.07 (0.53; 2.20)	0.84
Low HDL-cholesterol	0.67 (0.30; 1.49)	0.32	0.55 (0.25; 1.20)	0.13
*Model C*				
Age (years)	1.05 (1.02; 1.09)	0.003	1.05 (1.02; 1.08)	0.002
Gender (men)	0.50 (0.27; 0.92)	0.03	0.61 (0.34; 1.07)	0.09
Metabolic syndrome	1.53 (0.80; 2.92)	0.20	1.33 (0.72; 2.45)	0.37

Odds ratios are from multivariable logistic regression. ‘Obesity’ refers to increased waist circumference as defined by the IDF.

Univariate analysis of the components of the metabolic syndrome and osteoarthritis of the hip joint showed only borderline non-significant positive correlation of glucose levels with right hip degeneration (p = 0.05). None of the other components nor complete metabolic syndrome showed a significant increase of hip osteoarthritis prevalence (Table 2). Multivariable analysis demonstrated a significant increase of degeneration of the right hip in patients with elevated blood glucose (p = 0.02; Model B), while neither of the other components were associated with osteoarthritis (Table 3). The compound metabolic syndrome showed a tendency towards increased osteoarthritis of the right (OR: 1.33) and left (OR: 1.53) hip, however without being significant (p≥0.20). Additional analyses accounting for multiple testing revealed no significant association of any included risk factor with hip degeneration, except age (p = 0.003).

Testing the association of metabolic syndrome and its components with features of the OA MRI score showed that low HDL-c significantly reduced joint gap narrowing in both hip joints (p = 0.01). Elevated triglycerides significantly increased joint gap narrowing of the right hip, while elevated blood pressure significantly increased osteophytic lipping of the left hip (p≤0.04; Tables 4 and 5). Except for low HDL-c, the results were not significant when both joints were grouped together (p>0.08).

**Table 4:** Association of metabolic syndrome and its components with features of the OA MRI score for the right hip.

	Features of the OA MRI score					
Risk factors	Joint gap narrowing		osteophytic lipping		Subchondral changes	
	OR (95% CI)	p	OR (95% CI)	p	OR (95% CI)	p
Obesity	1.21 (0.73; 2.01)	0.47	0.74 (0.44; 1.25)	0.26	0.83 (0.34; 2.07)	0.70
Elevated BP	1.01 (0.62; 1.64)	0.96	1.46 (0.88; 2.40)	0.14	0.58 (0.20; 1.63)	0.30
Elevated glucose	1.56 (0.97; 2.53)	0.07	1.26 (0.77; 2.07)	0.35	1.22 (0.48; 3.11)	0.68
Elevated triglycerides	1.96 (1.10; 3.50)	0.02	1.38 (0.78; 2.45)	0.27	0.12 (0.01; 1.00)	0.05
Low HDL-cholesterol	0.41 (0.22; 0.79)	0.01	0.69 (0.36; 1.31)	0.26	0.47 (0.05; 3.98)	0.49
Metabolic syndrome	1.32 (0.84; 2.09)	0.23	1.31 (0.81; 2.12)	0.27	0.47 (0.16; 1.33)	0.15

Adjusted for age and gender. ‘Obesity’ refers to increased waist circumference as defined by the IDF.

**Table 5:** Association of metabolic syndrome and its components with features of the OA MRI score for the left hip.

	Features of the OA MRI score					
Risk factors	Joint gap narrowing		osteophytic lipping		Subchondral changes	
	OR (95% CI)	p	OR (95% CI)	p	OR (95% CI)	p
Obesity	0.94 (0.56; 1.57)	0.80	0.81 (0.47; 1.37)	0.43	0.41 (0.14; 1.22)	0.11
Elevated BP	1.19 (0.73; 1.93)	0.49	1.73 (1.04; 2.88)	0.04	0.49 (0.14; 1.76)	0.28
Elevated glucose	1.50 (0.93; 2.44)	0.10	1.24 (0.75; 2.04)	0.40	2.04 (0.67; 6.28)	0.21
Elevated triglycerides	1.27 (0.72; 2.24)	0.41	1.50 (0.84; 2.68)	0.18	0.28 (0.05; 1.64)	0.16
Low HDL-cholesterol	0.43 (0.23; 0.82)	0.01	0.57 (0.30; 1.09)	0.09	1.18 (0.21; 6.62)	0.85
Metabolic syndrome	1.21 (0.76; 1.92)	0.42	1.48 (0.91; 2.39)	0.11	0.54 (0.16; 1.76)	0.31

Adjusted for age and gender. ‘Obesity’ refers to increased waist circumference as defined by the IDF.

### Inter- and intrareader agreement

Interreader agreement for osteoarthritis of the right (K = 0.94) and left hip (K = 0.88) was almost perfect. The same was seen for the degree of the OA MRI score, with an almost perfect agreement for the right (K = 0.86) and left hip (K = 0.9). Furthermore, intrareader agreement was also almost perfect for the right (K = 0.97) and left hip (K = 0.96). The intrareader agreement for the degree of the OA MRI score showed almost perfect agreement for the right (K = 0.97) and left (K = 0.96) hip joint, respectively.

## Discussion

The results of our population-based cross-sectional study indicate that degeneration of the hip joint is not related to the compound metabolic syndrome but may be associated with elevated blood glucose levels. This is particularly relevant due to the increasing incidence of metabolic syndrome as well as its components, especially overweight and obesity as well as diabetes mellitus. Thus, understanding its impact on the musculoskeletal system in our steadily aging population is highly relevant.

In our study, blood glucose was a borderline none-dependent and a dependent factor for osteoarthritis of the right hip. None of the other singular component was significantly related to increased osteoarthritis of the hip. The compound metabolic syndrome showed a none significant tendency to increase osteoarthritis of both hips. These results are partially in line with previous studies which show that most singular components of metabolic syndrome as well as the compound metabolic syndrome have no impact on osteoarthritis of the hip joints [10, 27, 28], whereas other studies have shown, that prevalence of metabolic syndrome is higher in patients with osteoarthritis [29]. It has to be acknowledged, that most studies showing a positive association between metabolic syndrome or its singular components (e.g. elevated blood pressure, hypercholesterolemia and blood glucose levels) have studied osteoarthritis of the knee, but not the hip [5, 12, 30]. This might be explained due to the increased prevalence and radiological signs of knee osteoarthritis compared to hip osteoarthritis [31–33]. Furthermore, with being a weight bearing joint, the degenerative changes of the knee may primarily be caused by an increased mechanical load [8, 16]. This was recently described in a mouse experiment, where severely obese mice had increased cartilage damage compared to metabolic mice with milder adiposity [8]. In addition, a prospective cohort study with 62,661 subjects found no increased risk of total hip and knee replacement for metabolic syndrome or the singular components. However, elevated waist circumference and hypertension seemed to increase the risk for total knee replacement [9].

A recent meta-analysis of Louati et al. (2015) showed that diabetes mellitus was associated with osteoarthritis of the knee and hand, yet there was no significant relation for the hip joint [3]. Furthermore, a systemic analysis of 40 studies (21.299 participants) showed no evidence for diabetes as an independent risk factor for hip and hand osteoarthritis, but little evidence for knee osteoarthritis [27]. However, it should be stated that only about half of the evaluated studies used objective radiographic measurements [27].

Several studies in the literature proposed that osteoarthritis and hypertension share traditional risk factors (i.e. age, obesity). However, a specific relationship between the pathological pathways is still yet to be discovered [11]. A recent meta-analysis study by Zhang et al. demonstrated a significant impact of hypertension on radiographic as well as symptomatic osteoarthritis of the knee [11]. However, we were not able to confirm these findings for the hip which might be due to the evaluation of different joints or the relatively small sample size with solely Caucasian and central European participants.

Interestingly, Zhou et al. found an association with an increased risk of knee osteoarthritis in relation to elevated triglycerides and cholesterol levels [21], which could not be confirmed for the hand in a prospective study with only female participants. Though, higher HDL-C levels seemed to be protective, while elevated triglyceride levels seemed to be a risk factor for the development of hand osteoarthritis [22]. Our results were similar to the latter study, with no significant association of hip osteoarthritis and lipemia. This might be due to the European-based cohorts compared to a Chinese population as well as the different evaluated joints. However, HDL-C was not seen as protective as by Garcia-Gil et al., which might be due the solely female participants as compared to our study with predominantly male participants.

Another important aspect to be considered is the potential reverse association between osteoarthritis and metabolic syndrome, i.e. that osteoarthritis is a significant independent predictor for developing diabetes mellitus [17]. Furthermore, hip and knee osteoarthritis are also associated with increased cardiovascular risk [34], due to the limited mobility of osteoarthritic patients [14, 17, 34]. In addition, Hawker et al. described an osteoarthritis induced reduction in walking as a significant, potentially modifiable risk factor for complications of diabetes [14]. Although we did not test for this reverse association and a cross-sectional study limits a clear demonstration of cause and impact, a similar association may also be possible for this study. Our participants with metabolic syndrome had a significantly elevated BMI and were (non-significantly) less physically active compared to the controls, indicating that limited physical activity may also be related to metabolic syndrome.

It is well known that age predicts osteoarthritis of the hip joints independently [35], which was confirmed in this study as expected. Beyond this, McCarthy et al. demonstrated that age above 50 years and osteoarthritis is associated with an elevated risk of receiving hip endoprosthesis within the next two years [36].

Except for low HDL-c, the irregular findings concerning the association between metabolic syndrome and the features of the used OA MRI score may be solely by chance. In a prospective study with a follow-up of 10 years, Garcia-Gil et al. found no significant association regarding serum lipids and osteoarthritis of the hand. However, increased levels of HDL-c appeared to have a protective impact [22].

There are limitations to this study. First, this study cohort is a sample from a healthy general Caucasian population, therefore, a comparison with non-Caucasian population might be limited. Second, we had a relatively small sample size of participants with metabolic syndrome, its individual components and hip osteoarthritis. Therefore, larger clinical cohorts in defined patients with metabolic syndrome will be necessary in order to address these aspects. Furthermore, our participants with metabolic syndrome and its components were therapeutically well adjusted with their nutritional and/or medicational treatment. However, this cohort represents a homogenous general population which was randomly selected. Third, morphological changes to the femoral head and acetabulum (e.g. Cam-impingement, pincer-sign) were not evaluated due to a limited assessability on the whole-body MRI scans. Furthermore, specific medical history concerning the hip joint (e.g. pain or surgery) as well as clinical exams (e.g. range of motion) were not included in the study protocol. Lastly, the employed OA MRI score is based on the widely used Kellgren-Lawrence classification for radiographs, which has several limitations like inconsistency by the developing authors as well as the fact that the radiographic features are not specified in detail, resulting in a subjective driven classification.

## Conclusion

The compound metabolic syndrome showed no association with osteoarthritis of the hip joint. Age was the only parameter to be dependently and independently associated to osteoarthritis of both hip joints, while elevated blood glucose was independently associated with degeneration of the right hip joint.
